# Exploring Biodiversity and Arsenic Metabolism of Microbiota Inhabiting Arsenic-Rich Groundwaters in Northern Italy

**DOI:** 10.3389/fmicb.2019.01480

**Published:** 2019-07-02

**Authors:** Lucia Cavalca, Sarah Zecchin, Patrizia Zaccheo, Ben Abbas, Marco Rotiroti, Tullia Bonomi, Gerard Muyzer

**Affiliations:** ^1^Dipartimento di Scienze per gli Alimenti, la Nutrizione e l′Ambiente (DeFENS), Università degli Studi di Milano, Milan, Italy; ^2^Dipartimento di Scienze Agrarie e Ambientali – Produzione, Territorio, Agroenergia (DiSAA), Università degli Studi di Milano, Milan, Italy; ^3^Department of Biotechnology, Delft University of Technology, Delft, Netherlands; ^4^Department of Earth and Environmental Sciences, University of Milano-Bicocca, Milan, Italy; ^5^Microbial Systems Ecology, Department of Freshwater and Marine Ecology, Institute for Biodiversity and Ecosystem Dynamics, University of Amsterdam, Amsterdam, Netherlands

**Keywords:** arsenic, groundwater, arsenate-reducing bacteria, arsenite-oxidizing bacteria, sulfur bacteria, iron bacteria

## Abstract

Arsenic contamination of groundwater aquifers is an issue of global concern. Among the affected sites, in several Italian groundwater aquifers arsenic levels above the WHO limits for drinking water are present, with consequent issues of public concern. In this study, for the first time, the role of microbial communities in metalloid cycling in groundwater samples from Northern Italy lying on Pleistocene sediments deriving from Alps mountains has been investigated combining environmental genomics and cultivation approaches. 16S rRNA gene libraries revealed a high number of yet uncultured species, which in some of the study sites accounted for more of the 50% of the total community. Sequences related to arsenic-resistant bacteria (arsenate-reducing and arsenite-oxidizing) were abundant in most of the sites, while arsenate-respiring bacteria were negligible. In some of the sites, sulfur-oxidizing bacteria of the genus *Sulfuricurvum* accounted for more than 50% of the microbial community, whereas iron-cycling bacteria were less represented. In some aquifers, arsenotrophy, growth coupled to autotrophic arsenite oxidation, was suggested by detection of arsenite monooxygenase (*aioA*) and 1,5-ribulose bisphosphate carboxylase (RuBisCO) *cbbL* genes of microorganisms belonging to *Rhizobiales* and *Burkholderiales*. Enrichment cultures established from sampled groundwaters in laboratory conditions with 1.5 mmol L^-1^ of arsenite as sole electron donor were able to oxidize up to 100% of arsenite, suggesting that this metabolism is active in groundwaters. The presence of heterotrophic arsenic resistant bacteria was confirmed by enrichment cultures in most of the sites. The overall results provided a first overview of the microorganisms inhabiting arsenic-contaminated aquifers in Northern Italy and suggested the importance of sulfur-cycling bacteria in the biogeochemistry of arsenic in these ecosystems. The presence of active arsenite-oxidizing bacteria indicates that biological oxidation of arsenite, in combination with arsenate-adsorbing materials, could be employed for metalloid removal.

## Introduction

Natural contamination of groundwater with arsenic (As) represents a serious issue in both developing as well as many developed countries (Smedley and Kinniburgh, 2002). Moreover, in countries where groundwater represents the major source of drinking water, As poses problems to public health, such as in Bangladesh (Cavalca et al., 2013; Yunus et al., 2016). In Europe, As in drinking waters represents a risk for humans in different countries like Greece, Hungary, Romania, Croatia, Serbia, Spain (Katsoyiannis et al., 2015), and Ireland (McGrory et al., 2017). Particularly, in Italy As is present in groundwater at concentrations that are above the WHO limit of 10 μg L^-1^, due to geological history and substratum rock types, i.e., volcanic rocks in Central Italy and Pleistocene sedimentary rocks in Northern Italy (Mantelli et al., 1999; World Health Organization [WHO], 2001; Ungaro et al., 2008; Carraro et al., 2015; Peña Reyes et al., 2015). Specifically, in South-Eastern part of Lombardy (Po Plain) high levels of As can be measured in groundwaters characterized by low redox and oxygen content, high concentration of iron (Fe), manganese (Mn) and ammonium (NH_4_), and by the presence of peat (Rotiroti et al., 2014, 2015). In this area, drinking water is supplied through groundwater wells, thus posing a pollution problem of drinking water wells and subsequent health problems to the inhabitants of this area.

Redox processes govern As biogeochemistry of groundwater systems (“aquifers”). Anaerobic conditions leading to As solubilization are driven by the degradation of peat deposits and fuel reductive dissolution of As-bearing amorphous Fe (hydr)oxides by metal-reducing bacteria (Lear et al., 2007; Héry et al., 2008; Sutton et al., 2009; Cai et al., 2013; Herath et al., 2016). Microbial reductive processes are implicated in the dissolution of As from Fe, Mn, and aluminum minerals, either carried out by Fe- and to a lesser extent Mn-reducing bacteria (Islam et al., 2004; Luna et al., 2009), or by dissimilatory arsenate [As(V)]-reducing bacteria (DARB), fueled by the oxidation of organic substrates (Islam et al., 2004; Malasarn et al., 2004; Mladenov et al., 2010). Metal-reducing bacteria, like *Geobacter* present in Fe- and Mn-rich groundwater, play a role in releasing As to groundwater either by reducing As-bearing Fe-oxides (Giloteaux et al., 2013) or by reducing sediment-bound As(V) to As(III) (Héry et al., 2010). Similarly to *Geobacter*, also *Shewanella* (Dhar et al., 2011), *Sulfurospirillum* (Héry et al., 2008) and *Desulfuromonas* (Osborne et al., 2015) were found to promote Fe(III) reduction and As release from the sediment to the aquifer via dissimilative reduction processes. In anaerobic and microaerophilic environments, sulfur (S)-oxidizing bacteria can contribute to pyritic-S dissolution by the oxidation of solid S as sulfide, elemental S and thiosulfate (Kodama and Watanabe, 2003; Campbell et al., 2006; Handley et al., 2014; Thiel et al., 2019). On the other hand, sulfate reduction decreases the mobility of As by co-precipitation of sulfide with Fe and As (Kirk et al., 2004).

Once As is solubilized from sediments, it can undergo reductive detoxification (i.e., reduction of As(V) to the more mobile As(III)) conducted by microorganisms bearing the ARS operon (Andres and Bertin, 2016). Conversely, oxidation processes lead to immobilization of As on solid Fe-oxide by chemolithoautotrophic bacteria present in groundwater systems (Alfreider et al., 2009) and able to oxidize As(III) to As(V) (Garcia-Dominguez et al., 2008; Andreoni et al., 2012). In subsurface groundwater at circumneutral pH, Fe-oxidizing bacteria (FeOB) like *Gallionella ferruginea* (Katsoyiannis and Zouboulis, 2004; Gorra et al., 2012) and anaerobic nitrate-reducing/Fe-oxidizing bacteria contribute to the immobilization of As *via* their ability to form solid Fe (hydro)oxides minerals (Hohmann et al., 2010).

In subsurface groundwaters, complex bacterial communities were deciphered at the genome level, evidencing inter-organism interactions involved in ecosystem plasticity (Anantharaman et al., 2016). Bacterial communities in As-rich groundwater have been reported to be dominated by *Firmicutes, Alpha-, Beta-, Gamma-* and *Epsilonproteobacteria* (Bertin et al., 2011; Liao et al., 2011; Lin et al., 2012; Escudero et al., 2013; Héry et al., 2015; Wang et al., 2016; Crognale et al., 2017). In order to evidence the distribution, phylogeny and activity of As bacteria in groundwater, functional molecular markers have been applied in several groundwater systems (Escudero et al., 2013; Li et al., 2017; Wang et al., 2018). Arsenite-oxidizing bacteria (AOB) have been studied by analyzing the diversity of *aioA* genes, encoding the large subunit of As(III) oxidase (AioA) with different molecular approaches (Hamamura et al., 2009; Heinrich-Salmeron et al., 2011; Xiao et al., 2016; Crognale et al., 2017). This gene has been retrieved in natural surface waters characterized by As concentration ranging from 0.01 to 4 mg L^-1^ As (Escudero et al., 2013) and in groundwater with >1 mg L^-1^ As (Quéméneur et al., 2010). Genes coding for dissimilatory As(V) reductase (ArrA) were used for demonstrating the presence of As-releasing microbial processes in anoxic groundwater in the Coastal Plain in New Jersey (United States) with As ranging from 20 to 80 μg L^-1^ (Barringer et al., 2010) and in Chinese and Cambodian groundwater sediments (Héry et al., 2015; Li et al., 2017). As resistance by means of As(V) reduction has been investigated widely both in environmental samples as well as in bacterial strains isolated from groundwaters (Guo et al., 2015; Sarkar et al., 2016).

Previous studies focused on the microbiological characterization of As-rich groundwaters in South and South East Asia, including Bangladesh (Hassan et al., 2016; Sultana et al., 2017), China (Guo et al., 2015; Li P. et al., 2015; Wang et al., 2016), West Bengal (Osborne et al., 2015) and Taiwan (Das et al., 2016). Here, As pollution is more severe in shallow aquifers (generally < 50 m below surface) formed by Holocene sediments (Ravenscroft et al., 2009; Fendorf et al., 2010; Zheng et al., 2019). A similar situation has been observed in shallow aquifers in the Mississippi Delta in Southern Louisiana, where microbial respiration has been supposed to be responsible for As dissolution (Yang et al., 2014).

It has been postulated that groundwater As content declines with sediment age and it increases in concomitance with microbial degradation of fresher, and thus more reactive, organic matter (Postma et al., 2012; Stuckey et al., 2016). Conversely, in the Po Plain in Northern Italy, As pollution is more severe in intermediate aquifers (between 50 and 150 m below surface) lying on Pleistocene sediments (Rotiroti et al., 2014, 2015). Here, sediments and the buried organic matter (peat) driving As release to groundwater are older. Therefore, the above-mentioned relationship between groundwater As concentrations and sediment age/freshness of organic matter cannot be applied. A similar phenomenon has been observed in the Mahomet aquifer in Illinois, a Pleistocene glacial aquifer system (Kirk et al., 2004; Kelly et al., 2005).

Hydrogeological and microbial processes related to As dissolution in aquifers lying on recent Holocene sediments, like those in South and South East Asia, have been comprehensively characterized. At the best of authors’ knowledge, microbial characterization of aquifers in ancient floodplain areas, like those lying in the Po Plain at the foot of the Alps, is a novel aspect that cannot be found in previous works.

In this context, the present study aimed to decipher the microbial composition of groundwater residing on Pleistocene sediments in Northern Italy thus expanding knowledge on As dynamics in worldwide contaminated aquifers. Furthermore, the detection of microbial functionality toward As was investigated in order to evidence the potential to exploit resident bacterial populations for metalloid removal.

## Materials and Methods

### Hydrogeological Features and Physicochemical Characteristics of Groundwaters

Water samples were collected from six sites located in the province of Cremona (Lombardia, Northern Italy, see Supplementary Figure 1). Samples came from four public-supply wells of drinking water (samples A, B, D, and I), one monitoring well (sample L) and one biofilter unit (sample B-WW) (Table 1). The studied area hosts a multilayer system characterized by vertical alternations of sands (aquifer units) and silty clays (aquitard units), which features the whole lower Po Valley (Ori, 1993). Well A is located in an area closer to the Oglio River where aquifer units are considerably separated by silty clay layers. It taps two overlapping aquifer units separated by a ∼50 m thick silty clay layer. Well D is located in an area closer to the Po River, where sands are abundant and overlapping aquifer units are less separated. Wells B, I and L are located in areas equidistant from both the Po and Oglio Rivers and their hydrogeological features are intermediate with respect to those described for wells A and D. Well B taps a unique deep aquifer unit. Although well I has two screen intervals, it taps a unique aquifer unit; this aquifer is overlaid by a 13 m thick peaty clay layer. Sampling point L is a shallow piezometer tapping the unconfined aquifer (Rotiroti et al., 2014).

**Table 1:** Physicochemical properties of the studied water samples.

Municipality	Coordinates	Sample	Water type	Depth (m below surface)	Well screen interval^a^ (m below surface)	T(°C)	pH	Redox potential (mV)	Total hardness (mgL^-1^ CaC0_3_)	Dissolved components (μgL^-1^)									
										Organic C	S-S0_4_	P-PO_4_	N-NO_3_	N-NH_4_	Fe	Mn		As		
																	Total	As(III)	As(V)
Pescarolo	N45°ll′ 37.943′′ E10° 11′ 11.306′′	A	Public supply	210	97- 103	14.7	7.58	–113	282	2.11	267	165	685	2680	759	96.6	171	132	33
Pozzaglio	N45°12′ 1.335′′ E10°3′ 1.214′′	B	Public supply	201	151.4- 181.2- 194.9	16.3	7.62	–92	262	0.56	167	168	3.0	1231	262	70.6	24	17	4.6
S. Daniele Po	N45°3′ 49.572′′ E 10° 10′ 56.193′′	D	Public supply	189	168- 180	16.4	7.29	–120	n.d.	n.d.	<0.5	131	<0.5	1562	301	56.7	32	28	5.9
Malagnino	N45°8′5.176′′ E10°6′ 53.186′′	I	Public supply	164	131.5- 134.5	16.1	7.73	–140	260	0.56	<0.5	112	<0.5	1240	381	77.8	36	29	6.9
Derovere	N45°6′ 35.899′′ E 10° 14′ 52.717′′	L	Monitoring well	10	5-10^b^	14.0	7.17	–104	435	n.d.	4167	87	75.0	778	3198	112	53	47	7.3
Pozzaglio	–	B-WW	Biofilter	0	n.d.	16.0	7.63	+456	n.d.	n.d.	n.d.	n.d.	n.d..	n.d.	157	n.d.	22	0	24

*n.d. not determined; ^*a*^data from TANGRAM database (Bonomi et al., 2014); ^*b*^supposed well screen interval.*

### Sampling and Physicochemical Analysis of Environmental Parameters

Groundwater samples were collected by purging wells until groundwater temperature, pH, dissolved oxygen and redox potential (Eh) were stabilized. Samples were transported on ice to the laboratory and frozen at -20°C until analyses. All sample bottles and caps were acid-washed and autoclaved before use. pH, dissolved oxygen (DO) and Eh were measured within few hours after sampling, using a pH-meter PCE-228 (PCE Deutschland GmbH, Meschede, Germany), a portable dissolved oxygen-meter-HI 9146 (Hanna Instrument US Inc., Woonsocket, United States) and a mV-meter PCE-228 (PCE Deutschland GmbH, Germany), respectively. Total dissolved carbon was measured by applying the potassium dichromate standard method (ASTM D1252-06, 2012). Ammonium (NH_4_) and nitrate (NO_3_) were measured by flow injection analysis and spectrometric detection (FIAstar 5000 Analyzer, Foss Tecator, Denmark). Sulfate was analyzed combining the gravimetric and colorimetric methods according to Murphy and Riley (1962). Samples for As, Fe and Mn analysis were preserved by filtering (0.22 μm) and adding HNO_3_ to a final concentration of 2% (v/v) and the elements were measured by inductively coupled plasma mass spectrometry (ICP-MS, Agilent Technologies, United States), using a multistandard solution ranging from 0 to 1 mg L^-1^ (Agilent Technologies, United States). To determine inorganic As speciation, As(V) and As(III) were separated by filtration according to Kim et al. (2007), using WATERS Sep-Pak^®^ Plus Acell Plus QMA cartridge (Waters, MA, United States), and followed by ICP-MS analysis as described above.

### Nucleic Acid Extraction

The microbial biomass was collected from 25 L of water that was filtered over 0.22 μm hollow fiber filters (Mediakap-5, SpectrumLabs, United States) and stored at –20°C until nucleic extraction. Biomass was detached from the filter by thoroughly rinsing with 10 mL of 0.2% (w/v) sodium pyrophosphate, and collected from the cartridge with a syringe. The suspension was centrifuged (10,000 rpm for 25 min at 10°C) and total DNA was extracted from the pellets using the PowerSoil^®^ DNA Isolation Kit (MO-BIO Laboratories, Inc., Carlsbad, CA, United States), with two additional lysing steps at 65°C for 30 min, and 90°C for 5 min. The DNA from the enrichment cultures was isolated with UltraClean^TM^ Microbial DNA Isolation Kit (MO-BIO Laboratories). The extracted DNA was visualized on agarose gel, and the quality and quantity were determined with a ND-1000 spectrophotometer (Nanodrop Inc., Wilmington, DE). PowerWater^®^ RNA Isolation Kit (MO-BIO Laboratories), was used for RNA extraction from groundwater samples, according to manufacturer’s instructions. Despite different attempts, RNA extractions were unsuccessful.

### 16S rRNA Gene Amplicon Sequencing

Barcoded fragments (∼ 570 bp), spanning the V4 and V6 hypervariable regions (*Escherichia coli* position 530–1100) of the 16S rRNA gene were amplified from the groundwater DNA extractions using universal bacterial primers 530F (5′-GTGCCAGCMGCNGCGG-3′) and 1100R (5′-GGGTTNCGNTCGTTR-3′), fused to 454 A and B adaptors, respectively (De León et al., 2012). PCR reactions for each sample were carried out in triplicate and then pooled together for pyrosequencing with Life Sciences 454 Genome Sequencer FLX platform (Roche, Switzerland) according to standard protocols. A negative control (blank) was included for each PCR reactions. A 100 ng (1 μL) aliquot of each DNA sample was used for a 50 μL PCR reaction. Taq Master Mix Kit (Qiagen) was used for PCR under the following conditions: 94°C for 3 min followed by 32 cycles of 94°C for 30 s; 60°C for 40 s; and 72°C for 1 min; and a final elongation step at 72°C for 5 min.

Quality treatment was performed by using Pyrotagger (Kunin and Hugenholtz, 2010), removing low quality data. QIIME was applied as sequence quality filter to the original 16S rRNA gene sequence dataset based on the sequence quality log file (Caporaso et al., 2010). Sequences shorter than 200 nucleotides, those with one or more ambiguous bases and those that received a quality score lower than 25 were eliminated. After this, an average of 3000 reads per sample were considered. QIIME was used for phylogenetic analysis of 16S rRNA sequences. Bacterial operational taxonomic units (OTUs) were defined at a value of 97% similarity of the 16S rRNA gene sequences. The number of sequences present in each sample after applying filtering is shown in Supplementary Figure 4A.

Unique pyro-sequences of 16S rRNA genes were aligned using the “align.seqs” command and the Bacterial SILVA SSU Ref database (Release 119). Sequences were taxonomically classified by the RDP-II Naïve Bayesian Classifier using a 60% confidence threshold against the SILVA Database. A phylogenetic tree was constructed using the FastTree with Kimura’s two-parameter model (Price et al., 2009). To investigate patterns in alpha diversity, rarefaction analysis was performed and collector’s curves were calculated (Supplementary Figure 4B), including the Chao1 richness estimator (Chao, 1984) and the Shannon Diversity Index. To investigate patterns in beta-diversity, pairwise distances between bacterial communities were calculated with the UniFrac distance matrix (Lozupone and Knight, 2005). Predictive microbial As, Fe, and S processing profiling within the 16S Amplicon sequencing library was performed according to Zecchin et al. (2017). The reference database used for this analysis included recent updates on literature published within the end of 2018.

The microbial communities of groundwater samples were also analyzed by denaturing gradient gel electrophoresis (DGGE) analysis of 16S rRNA genes using primers Bac341F-GC and Bac907rM according to Schäfer and Muyzer (2001). Details of the PCR reactions, concentration of the reagents, thermal protocol, electrophoresis, and data analysis are described in Supplementary Material Section “Experimental Conditions.”

### qPCR of Functional Genes

The presence of As and RuBisCO genes was tested and quantified by real time qPCR reactions. Particularly, genes for arsenite oxidase (*aioA*), arsenate reductase (*arsC*), arsenate respiratory reductase (*arrA*), and for the large subunit of ribulose-1,5-bisphosphate carboxylase/oxygenase (RuBisCO) Type-I (*cbbL*) were investigated.

Amplification of arsenite oxidase gene *aioA* was performed with primers aoxBM1-2F and aoxBM3-2R according to the protocol of Quéméneur et al. (2008). Amplification of arsenate reductase gene *arsC* was conducted with primers P52F and P323R according to Bachate et al. (2009). Amplification of arsenate respiratory reductase *arrA* was performed with primers ArrAfwd and ArrArev according to the protocol of Malasarn et al. (2004). Amplification of the gene coding for the large subunit of RuBisCO Type-I (*cbbL*) was performed with the primers RBCO-1Cf and RBCO-1Cr according to the procedures of Alfreider et al. (2009). PCR reactions were performed in a final volume of 25 μL containing: 2 μL of template, 0.2 mmol L^-1^ of dNTPs, 1.75 mmoL l^-1^ of MgCl_2_, 0.5 μmol L^-1^ of each primer, 1.5 U of *Taq* polymerase and 1X PCR buffer. Amplification protocols used for *aioA, arsC, arrA* and *cbbL* gene amplifications were the same as reported by the authors. All the reagents were from Invitrogen. PCR reactions were carried out using the T-Gradient apparatus (Biometra, Germany). PCR products were checked on a 1.5% (w/v) agarose gel containing GelRed^TM^ stain (Biotium) at 0.01% (v/v) and visualized using a Bio-Rad Gel Documentation System (Bio-Rad).

The same targets were quantified in the samples by Real Time quantitative PCR (RT-qPCR). To quantify *aioA* genes, primers aoxBM1-2F and aoxBM2-1R were used, whereas for *arsC* and *arrA* quantification the primers were the same used for PCR amplification. Each reaction mixture included 10 μL of 2x SsoFast Evagreen Supermix (Bio-Rad, Hercules, CA), 0.2 ng μL^-1^ of each primer for *aioA* and *arsC* and 0.5 ng μL, and 2 μL of template DNA in a total volume of 20 μL. The thermal protocols used for the amplifications were set up according to Quéméneur et al. (2010) for *aioA* and to Bachate et al. (2009) for *arsC.* Amplification of *arrA* was carried out with the following protocol: 95°C for 10 min, 40 cycles including 95°C for 30 s, 55°C for 1 min and 72°C, and final step for melting curve generation with 55°C for 30 s followed by temperature increasing steps of 0.5°C s^-1^ each until 95°C. Genomic DNA isolated from *Achromobacter* sp. strain 1L was used to generate the standard curve for *aioA* quantification. To obtain the standard curve for *arsC* quantification, DNA isolated from *Bacillus licheniformis* strain SeaH-As1w was used. The standard curve for *arrA* quantification was generated with DNA isolated from *Desulfitobacterium hafniense* strain DCB-2.

### Clone Library Preparation

The purified PCR products were ligated into the pCR2.1-TOPO vector and transformed into TOP10 chemically competent *E. coli* cells according to the manufacturer’s protocol (Invitrogen, Carlsbad, CA). The transformed cells were plated on Luria-Bertani (LB) agar plates containing 100 μg mL^-1^ of ampicillin, 80 μg mL^-1^ of 5-bromo-4-cholo-3-ondolyl-b-D-galactopyranoside (X-Gal). After an overnight incubation at 37°C, white transformants were transferred to LB ampicillin (100 μg mL^-1^) agar plates and cultured overnight. To ensure the presence of the insert, PCR amplification was performed directly on the colonies by using specific primer pair for each target. The plasmids from a random selection of positive clones were purified and sequenced using M13 universal primers (Invitrogen) and the Taq Dye-Deoxy Terminator Cycle Sequencing kit (Life Technologies Co., Carlsbad, California, United States). The forward and reverse sequences for each target were obtained loading the respective samples on an ABI 310 Genetic Analyzer (Life Technologies Co., United States).

### Comparative Sequence Analysis

Nucleotide sequences of *aioA, arrA, arsC*, and *cbbL* genes obtained from clone libraries were manually edited, translated into amino acid sequences and searched for closely related amino acid sequences in GenBank database (BlastX). Obtained amino acid sequences and reference sequences for each target were aligned with ClustalX, and trees were built with MEGA 6 using the neighbor-joining distance method based on p-distance (Tamura et al., 2007). A total of 1000 bootstrap replications were calculated.

### Selection of Arsenic-Transforming Bacterial Cultures

Microorganisms involved in As cycle (i.e., autotrophic and heterotrophic As(III)-oxidizing bacteria (AAOB and HAOB), detoxifying As(V)-reducing bacteria (ARB), and dissimilatory As(V)-reducing bacteria) were enriched from groundwater samples. To set up the enrichments, BBWM medium (Corsini et al., 2014) added with 1.5 mmol L^-1^ sodium As(III) (NaAsO_2_) or with 15 mmol L^-1^ sodium As(V) (Na_2_HAsO_4_⋅7H_2_O) was prepared. Sodium lactate (NaC_3_H_5_O_3_) was added (0.4 mol L^-1^) to DARB, HAOB and to ARB enrichments, and omitted to AAOB enrichments. Water samples and culture medium were mixed on a 1:1 proportion and incubated at 28°C under shaking at 150 rpm (AAOB, HAOB and ARB). Set up of DARB enrichments were performed under anaerobic conditions in an anaerobic hood. Arsenic transformation within the enrichments were quantified by permanganate colorimetric analysis performed according to Salmassi et al. (2002) and Dhar et al. (2004).

## Results

### Physicochemical Characteristics of Groundwater Samples

The physicochemical characterization of water samples is described in Table 1. All groundwater samples were anoxic to mildly oxic, with Eh values ranging from -92 mV in samples B to -140 mV in samples I, whereas Eh in the biofilter unit B-WW was +456 mV. All samples were oligotrophic, with a maximum value of organic carbon of 2.11 μg L^-1^ measured in sample A. The pH was circumneutral in all samples. Arsenic concentrations ranged from 22 μg L^-1^ in biofilter sample B-WW to 171 μg L^-1^ in public supply well A (Table 1). In all samples, As(III) was the dominant As species. In sample B-WW, As content was similar to that of the original groundwater (site B), with As(III) being totally oxidized to As(V). Organic contaminants (i.e., aliphatic or aromatic hydrocarbons) and heavy metals (i.e., cadmium, mercury, selenium, vanadium, and antimony) were absent in all water samples.

### Bacterial Community Structure in Groundwater Samples

Barcoded pyrosequencing of 16S rRNA genes (16S Amplicon sequencing) was applied to obtain a detailed description of microbial community composition of the 6 samples.

**FIGURE 1:**
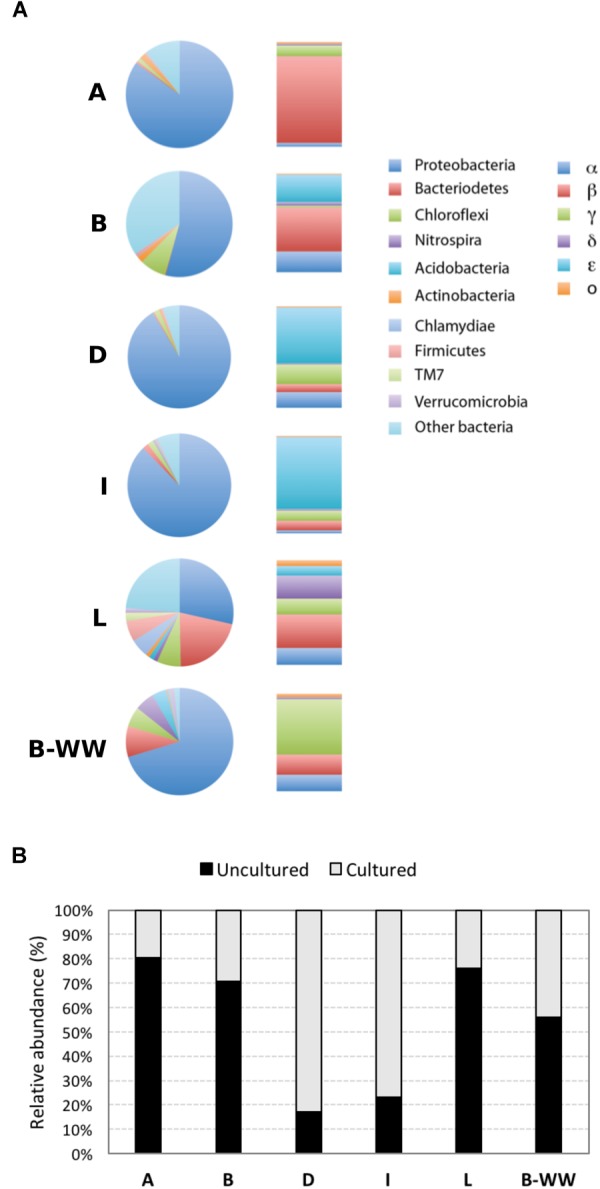
Microbial communities in groundwaters sampled from four drinking water public supplies (A, B, D, and I), one monitoring well (L) and one biofilter unit (B-WW), analyzed by 16S rRNA gene amplicon sequencing. Colored pie charts and bar graphs **(A)** show the percentage at the phylum level and the percentage of the different classes within the *Proteobacteria*, respectively. Gray-scale bars **(B)** represent the percentage of sequences of uncultured vs. cultured species retrieved in the water samples.

The rarefaction curve approximated toward a plateau after ∼3000 OTUs were sequenced, indicating that enough sample coverage was obtained in this study (Supplementary Figures 4A,B). The species richness estimator Chao1 and pairwise distances values showed that the samples had different levels of diversity (Supplementary Table 3). Samples formed three separate clusters: public supply wells, biofilter water and monitoring well, with the last one showing a high differentiation, as evidenced by the topology of the weighted UniFrac UPGMA tree (Supplementary Figure 5). Taxonomic classification indicated that 21 phyla of Bacteria and Achaea were present in the 6 samples. In samples A, B, L, and B-WW, 80, 70, 76, and 55%, respectively, of the reads were classified as uncultured taxa, at all taxonomic levels. In samples D and I, the same accounted for 17 and 23%, respectively (Figure 1B). *Proteobacteria* was the dominant phylum, followed by *Bacteroidetes* and *Chloroflexi* in most of the samples. *Firmicutes, Nitrospira, Clamydiae*, and *Acidobacteria* were less abundant, although in some wells represented more than 4% of the community (Figure 1A and Supplementary Figure 5). Within the *Proteobacteria, Betaproteobacteria* dominated in samples A and B (70.1 and 23.8%, respectively), *Epsilonproteobacteria* predominated in samples I and D (64.8 and 50.2%, respectively), and *Gammaproteobacteria* predominated in the biofilter sample W (40.0%) (Figure 1A). The most abundant *Alphaproteobacteria* were members of the genera *Sphingopyxis* in sample D (8.8%) and *Sphingomonas* in sample B (7.7%). The main populations of *Betaproteobacteria* were *Thiobacillus* (5.4%, in sample W) and *Methylophilus* (4.5 and 3.0% in samples A and W, respectively). Within *Epsilonproteobacteria* the only species retrieved was *Sulfuricurvum kujiense*, which dominated in four out of six groundwater samples and represented 64.8, 50.2, 14.7, and 2.6% of the bacterial community in samples I, D, B, and L, respectively. Within *Gammaproteobacteria, Thiotrix* (16.3%, in sample B-WW), *Pseudomonas* (15.1%, in sample D), *Azotobacter* (5.6%, in sample A) and *Methylomonas* (3.5%, in sample I) were the most representative genera.

**FIGURE 2:**
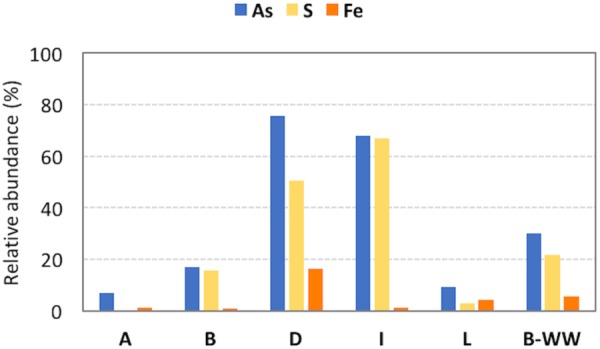
Relative abundances of microorganisms in the 16S rRNA amplicon sequencing library potentially involved in arsenic (As), sulfur (S) and iron (Fe) cycles in the different samples. The vast majority of S cycle-related species belong to *Sulfuricurvum* spp. in groundwaters B, D and I and to *Thiothrix* spp. in biofilter unit B-WW.

Among the retrieved genera, those documented to be involved in As resistance and oxidation, together with S oxidation, were dominant. In these planktonic communities, Fe-cycling bacteria were present although less abundant with respect to S bacteria (Figure 2, Supplementary Table 4, and Supplementary Figure 6). Inferred functionalities of these microbial genera are presented in the Discussion section.

Data from DGGE analysis (Supplementary Figure 2) performed on the same samples confirmed the presence of a high percentage of uncultured species (Supplementary Table 1), most of which related to S-oxidizing microorganisms (Supplementary Figure 3 and Supplementary Table 2).

### Diversity of Arsenic Genes in Groundwaters

In groundwater DNA As-cycling bacteria were quantified and depicted by genetic biomarkers (Table 2). The most abundant were *aioA* and *arsC*, with the highest values measured of 4.2 × 10^2^ and 6.8 × 10^2^ gene copies L^-1^, respectively. *ArrA* were found only in sample A in low amount (1.8 10^1^ copies L^-1^). In sample D, the amplification of any functional gene from the DNA was not possible, despite different attempts (Table 2).

**Table 2:** Biomarkers quantification (gene copies per L) of arsenic cycle genes and for chemoautotrophy in groundwater DNA.

Sample	Arsenic genes			RuBisCO gene
	*aioA*^∗^	*arsC*	*arrA*	*cbbL*
A	4.2 ⋅10^2^	–	1.8⋅10^1^	–
B	1.3 ⋅10^1^	–	–	+
D	–	–	–	–
I	1.1 ⋅10^1^	6.8 ⋅10^2^	–	+
L	1.9 ⋅10^2^	2.7 ⋅10^2^	–	–
B-WW	1.7 ⋅10^1^	–	–	–

*^∗^*aioA*, As(III) oxidase gene; *arsC*, detoxification As(V) reductase gene; *arrA*, dissimilatory As(V) reductase gene; *cbbL*, 1,5 ribulose bisphosphate carboxylase gene; +, positive PCR amplification; -, no PCR amplification.*

**FIGURE 3:**
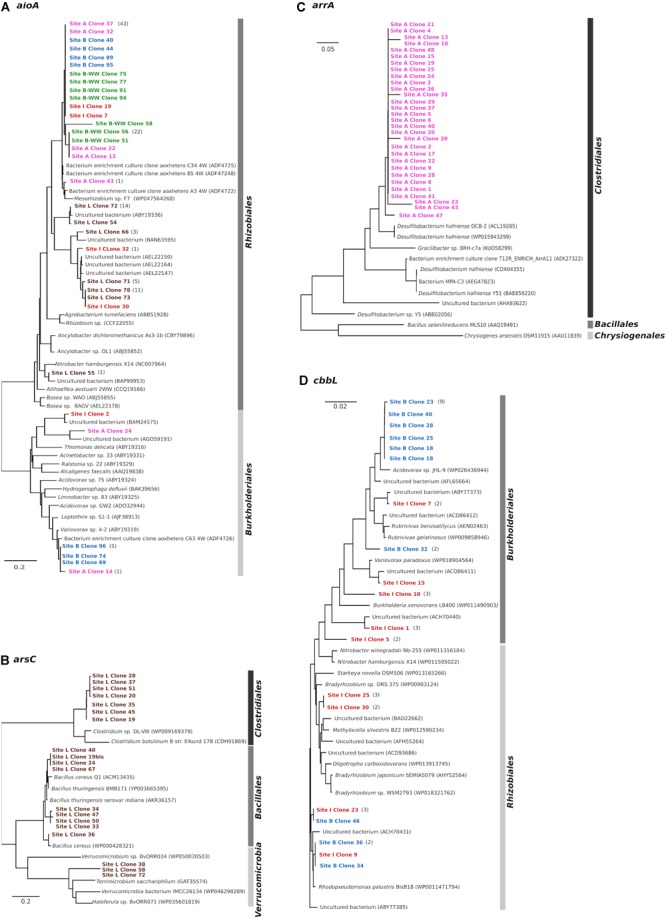
Phylogenetic analysis (neighbor-joining tree) of deduced amino acid sequences of *aioA* **(A)**, *arsC* **(B)**, *arrA* **(C)** and type I RuBisCO *cbbL* **(D)** genes cloned from arsenic-contaminated aquifers and sequences from the NCBI database. As outgroup for the calculations, the AioA sequence of *Hoeflea phototrophica* DFL-43, the ArsC sequence of *Achromobacter xylosoxidans* A8, the ArrA sequence of *Shewanella* sp. ANA-3 and the CbbL sequence of *Ancylobacter dichloromethanicus* As3-1b were used. The scale bar represents a difference of 0.2 nucleotides per position for AioA and ArsC, of 0.05 for ArrA, and of 0.02 nucleotides per position for CbbL.

*AioA* clone libraries constructed from five water samples contained 140 clones with expected insert size of 1111 bp. Translated amino acid sequences were grouped into two major clusters belonging to *Alphaproteobacteria* and *Betaproteobacteria*, regardless the origin of the water (Figure 3A). The cluster associated to *Alphaproteobacteria* exclusively included members of the order *Rhizobiales* (95–99% identity). Clones from site A, B, B-WW, and I were similar to the AioA sequence of uncultured bacteria from an As-contaminated soil (97–98% identity with ADF47224, ADF47258, ADF47248, Sultana et al., 2012). Sequences obtained from site I and from site L were related to uncultivated bacteria from different As-contaminated mines or sediments (95–99%, Quéméneur et al., 2008; Heinrich-Salmeron et al., 2011; Yamamura and Amachi, 2014). Finally, clone 55 from site L formed a cluster with the *aioA* of an uncultivated bacterium (97% to BAP99953) from enrichment cultures with As(III)-spiked paddy soil (Dong et al., 2016) and with the sequence of *Nitrobacter hamburgensis* X14, a facultative chemolithoautotroph bacterium (Muller et al., 2007). The cluster associated with *Betaproteobacteria* was related (81–96% identity) to members of the order *Burkholderiales*. Clone 14 from site A and clones 69, 74, and 96 from site B had 96% similarity to As(III) oxidases of *Variovorax* sp. 4-2 (ABY19319) which was isolated from a laboratory reactor for biological As(III) oxidation (Battaglia-Brunet et al., 2002), and of an uncultivated bacterium (AGO59191) from As-contaminated soil (Sultana et al., 2012). Finally, clone 24 from site A, closely related to the As(III) oxidase of an uncultured bacterium from As-rich geothermal water (Jiang et al., 2014), and clone 55 from site L, closely related to the *aioA* of an uncultivated bacterium from sediments (Yamamura and Amachi, 2014), formed a cluster with *Thiomonas delicata* (ABY19316).

Clone libraries constructed with *arsC* amplicons from sample L contained 20 clones. The translated amino acid sequences of *arsC* gene were grouped into two major clusters: *Firmicutes* and *Verrucomicrobia* (Figure 3B). The cluster associated with *Firmicutes* was related to members of the orders *Bacillales* and *Clostridiales* (78–99% identity). The *Bacillales*-affiliated group was most closely related to the ArsC of several *Bacillus* species which were isolated from a deep-subsurface oil reservoir in the Daqing oil field in north-eastern China and from a soil in the United States (Xiong et al., 2009; Li Q. et al., 2015), whereas the *Clostridiales*-affiliated group was closely related to the ArsC of *Clostridium* species isolated from Pacific sediments and from an anaerobic sludge (Stringer et al., 2013; Taghavi et al., 2013). A second cluster was associated to ArsC of different members of *Verrucomicrobia* which were isolated from different environments, such as lake, roots and rice paddy soil (Qiu et al., 2014).

Clone libraries constructed with *arrA* amplicons from sample A resulted in 30 clones. They formed one cluster with the highest similarity to the ArrA sequences of *D. hafniense* strains (91% sequence similarity, Figure 3C), known anaerobic bacteria capable of dehalogenation and metal reduction (Pérez-Jiménez et al., 2005; Mumford et al., 2013).

### Diversity of RuBisCO Genes in Groundwater Samples

The presence of chemolithoautotrophic strains in the planktonic bacterial communities of two aquifer samples B and I was evidenced by the amplification of the *cbbL* gene, coding for the large subunit of ribulose-1,5-bisphosphate carboxylase/oxygenase (RuBisCO) Type-I (Table 2). The *cbbL* clone libraries from these two samples contained 40 clones. Phylogenetic analysis clearly separated the *cbbL* sequences into *Alpha-* and *Betaproteobacteria* (Figure 3D). The cluster associated with *Alphaproteobacteria* was related to members of the order *Rhizobiales* (96–98% identity). Two clones from site B and one clone from site I formed a group together with RuBisCO sequences of an uncultivated bacterium from tar-oil-contaminated porous aquifer (96% to ACH70431, Kellermann et al., 2012) and of a strain of *Rhodopseudomonas palustris* BisB18 isolated from freshwater sediment (96% to WP0011471794, Oda et al., 2008). Two clones from site I were clustered with members of *Beijerinckiaceae* and *Bradyrhizobiaceae* families. Finally, clones 46 from site B and 23 from site I were not clearly affiliated to other RuBisCO sequences and showed the highest homologies (96%) with *cbbL* genes of uncultured bacteria reported in Alfreider et al. (2009). The cluster associated with *Betaproteobacteria* was related to members of the order *Burkholderiales* (95–100% identity). Six out of 10 clones from site B formed one group with 100% similarity to the CbbL sequence of *Acidovorax* sp. JHL-9 (WP02643944), an autotrophic H_2_-oxidizing, nitrate-respiring bacterium isolated from a subsurface oxic-anoxic transition zone (Lee et al., 2015). Clones 1, 7, and 15 from site I had high similarity to CbbL of different uncultured *Betaproteobacteria* from tar-oil-contaminated aquifer (99% to ACH70442), groundwater (98% to ABY77373) and from grassland soil (95% to ACD86411), respectively (Alfreider et al., 2009; Videmsek et al., 2009; Kellermann et al., 2012).

### Arsenic-Transforming Bacterial Cultures

The presence of As-transforming bacteria in groundwaters was confirmed by enrichment cultures after multiple transplants on As(III) or As(V) (Table 3).

**Table 3:** Percentage (%) of arsenic transformation in the enrichment cultures.

Sample ID	As(V)-resistant bacteria		As(V)-respiring bacteria	Heterotrophic As(III)-oxidizing bacteria		Autotrophic As(III)-oxidizing bacteria	
	Reduction (%)	*arsC* gene	Reduction (%)	Oxidation (%)	*aioA* gene	Oxidation (%)	*aioA* gene
A	15	+	0	10	–	0	–
B	0	–	0	0	–	0	–
D	37	+	0	10	–	0	–
I	32	–	0	20	–	60	+
L	35	+	0	10	+	65	+
B-WW	40	–	0	100	+	100	+

Arsenic resistance by means of heterotrophic As(V) reduction and As(III) oxidation was retrieved in most of the samples. On the contrary, As(V) dissimilative reduction was not displayed in any groundwater sample. Under chemolithoautotrophic conditions, As(III) was oxidized in samples I, L, and B-WW. The culture from aerobic biofilter B-WW was the most active one with respect to cultures from groundwaters for all the As transformations. PCR amplifications of *ars*C and *aio*A genes confirmed the presence of As-transforming bacteria (Table 3).

Enrichment cultures where As metabolisms were evident, were subjected to DGGE analysis which resulted in the isolation of 29 successfully sequenced bands (see Supplementary Tables 5–7). Autotrophic As(III)-oxidizing enrichments (Supplementary Table 5), were composed of *Hydrogenophaga* sp. strains, previously reported to be autotrophic As(III) oxidizers (Cavalca et al., 2013) and *Acinetobacter* sp., *Acidovorax* sp. and *Exiguobacterium auranticum* strains previously reported to be heterotrophic As(III) oxidizers (Cavalca et al., 2013; Pandey and Bhatt, 2016). These species are common inhabitants in Chinese high As aquifers (Li et al., 2013; Li P. et al., 2015; Wang et al., 2016). For other species retrieved in autotrophic consortia (i.e., *Sphingopyxis chilensis, Luteimonas aestuarii*, and *Lysobacter capsici*), the oxidative activity has never been demonstrated, although in GenBank several As gene sequences are deposited from the genome of related species, including genes for As(III) oxidation. In the heterotrophic As(III)-oxidizing cultures (Supplementary Table 6), several As(III) oxidizers were found, including a close relative of *Delftia* sp., which was shown to be a facultative chemolithoautotrophic As(III) oxidizer (Sultana et al., 2017). In As(V) reducing enrichments most of the strains already retrieved in As(III) oxidizing cultures were detected, with the exception of *Microbacterium hydrocarbonoxydans* and *Rhodococcus ruber* strains (Supplementary Table 7). All were documented to be resistant to As(V) or to carry As genes in their genome.

## Discussion

Within the studied area, the content of sand and silt clay varies from the Po River in the Southwest, where sands are prevalent and thus overlapping aquifer units are less separated, to the Oglio River in the Northeast, where silts and clays are abundant generating a high degree of separation between overlapping aquifer units (Beretta et al., 1992; Vassena et al., 2012; Rotiroti et al., 2015). Deeper aquifer units (generally 30 m below the surface) are confined and characterized by extended groundwater circulation, and thus, by longer groundwater residence time. This aspect also influences groundwater chemistry: indeed, longer residence time and no or limited surface infiltration favor reducing environments, which promote the mobilization of As, Fe, Mn, and NH_4_ driven by the degradation of peat incorporated into semi-permeable silt and clay aquitards (Francani et al., 1994; Zavatti et al., 1995; Carraro et al., 2013; Rotiroti et al., 2014). The peak of As release is expected in intermediate aquifers (generally between 50 and 150 m below surface) since here: (i) reduction of Fe-oxides likely favors the release of As, and (ii) co-precipitation of dissolved As with sulfides is not yet prevailing (Carraro et al., 2015; Rotiroti et al., 2015).

As described by high throughput sequencing, a high number of uncultured species was present in most study sites, evidencing that these environments are still largely unexplored. Species composition in As-affected water samples of Lombardia, resembled those previously described in other planktonic communities of uncontaminated waters in the Netherlands (Roeselers et al., 2015), of metal-contaminated groundwater in the United States (Hemme et al., 2010) and of high As aquifer in Bangladesh (Legg et al., 2012).

A complete C cycle is supported in these oligotrophic environments by the presence of a large number of autotrophic and heterotrophic bacteria. In most samples, autotrophic metabolism could be inferred by different strategies like autotrophic As(III) oxidizing enrichment cultures, PCR amplification of the *cbbL* gene coding for RuBisCO as well as by detection of autotrophic genera in the 16S Amplicon data. Dissimilative nitrate respiration was largely represented by the presence of *Sulfuricurvum, Denitratisoma, Geobacter, Acidovorax*, and *Dechloromonas* genera, which couple S, Fe(II) and As(III) oxidation to NO_3_ reduction (Finneran et al., 2002; Sun et al., 2009; Carlson et al., 2013; Chakraborty and Picardal, 2013). Nitrogen fixation was represented by a large proportion of *Alphaproteobacteria* of the order *Rhizobiales*, which was also evidenced by *cbbL* gene detection of the *Azotobacter* genus.

Peculiar of four (I, D, B, and L) out of five studied water samples was the high abundance of *Epsilonproteobacteria* represented by the chemolithoautotrophic S-oxidizing genus *Sulfuricurvum* sp., *S. kujiense* has been detected in terrestrial sulfidic caves (Porter and Engels, 2008) and its predominance was observed in formation waters of oil sands reservoir (Hubert et al., 2012) and in aquifer sediments minimally impacted by residual contamination of uranium and vanadium (Han et al., 2012). High abundance of members of the genus *Sulfuricurvum* indicates that, in addition to Fe-cycling, chemolithoautotrophic S oxidation at the expense of nitrate or oxygen could be an important process in aquifers of Northern Italy, as in aquifers and peatland freshwaters previously analyzed (Haaijer et al., 2008; Handley et al., 2014). Recently, a large proportion of a single uncultured *Sulfuricurvum* species was inferred to perform C and N fixation on the basis of groundwater metagenomes (Handley et al., 2014; Anantharaman et al., 2016). Considering the large abundance measured in Northern Italian groundwaters, this species might play a pivotal role in these ecosystems.

### Arsenic Cycle

Dissimilative As(V) reduction seemed to be less represented in the aquifer communities as typical As-respiring bacteria were poorly detected, differently from South and South East Asia (Osborne et al., 2015; Das et al., 2016; Gnanaprakasam et al., 2017). Nevertheless, *arrA* gene belonging to *D. hafniense* highlighted the presence of this function in sample A. In this sample, DOC (2.11 μg/L) and As content (171 μg L^-1^) were the highest with respect to other samples. In this confined aquifer, the mobilization of As(V) from sediments could be linked to the degradation of peat incorporated into semi-permeable silty and clayey aquitards. DARB were not enriched from samples, indicating either their absence/low abundance or possible failures in the cultivation strategy.

According to both molecular biomarkers and enrichment cultures, the ARS detoxification system was more represented than the dissimilative As(V) reduction, in accordance with investigations in West Bengal (Paul et al., 2015) and China (Li et al., 2017) groundwaters. *ArsC* genes detected in the monitoring well sample L belonged to *Bacillus, Clostridium* and *Verrucomicrobium* strains in accordance with their presence in bar-coding libraries (0.4, 0.6 and 0.2%, respectively), as previously reported for different environments (Cavalca et al., 2013). These bacteria are responsible of the cycling of soluble As fractions, as As(V) reductases encoded by *arsC* genes are unable to reduce adsorbed As (Macur et al., 2004) and might be relevant for As(V) reduction to As(III) in the planktonic bacterial communities described in the present study. The *Sulfuricurvum* genus, dominant in 4 samples, is characterized by the presence of *arsC* in the genome (Han et al., 2012).

*AioA* genes coding for arsenite oxidase are present in genomes of the chemolithoautotrophic bacteria retrieved in the studied sites, such as *Nitrospira, Alphaproteobacteria* (*Rhizobiales, Rhodobacterales*), *Betaproteobacteria* (*Acidovorax, Hydrogenophaga, Nitrosospira*), already detected in As(III)-rich groundwaters of Bangladesh (Hassan et al., 2016) and China (Li et al., 2013; Li P. et al., 2015). The presence of this metabolism was consistent with the phylogeny of *aioA* genes retrieved in the environmental DNA, as well as with the enrichment of As(III) oxidizing bacteria.

In some of the retrieved bacterial species, a chemolithoautotrophic metabolisms with As(III) as electron donor can be postulated. In fact, *cbbL* gene sequences belonging to *Acidovorax* sp., *Variovorax* sp. and *Burkholderia* sp. were detected. These microorganisms have been demonstrated to perform As(III) oxidation (Hamamura et al., 2009; Cavalca et al., 2013), while for other species retrieved in the clone library, such as *Oligotropha carboxidovorans, Bradyrhizobium japonicum* and *Rhodopseudomonas palustris*, only protein sequences for As(III) oxidases are deposited in the NCBI database.

The presence of As(III)-oxidizing bacteria in the studied sites was confirmed *in vivo* by enrichment cultivation. These microorganisms could be successfully employed for bioremediation purposes, as previously shown for aquifers and soils (Corsini et al., 2015; Katsoyiannis et al., 2015; Karn and Pan, 2017).

Our analysis indicated that bacteria present in groundwaters were possibly involved in methylation of inorganic As, such as *Nitrosomonadales, Desulfovibrio, Methanobacterium* and *Clostridium*, although this metabolism was not investigated in the present study. Under subsurface conditions, the biotic conversion of inorganic to organic forms of As by arsenite methyltransferase ArsM has been recently demonstrated (Maguffin et al., 2015; Wang et al., 2018).

### Fe and S Cycles

Directly linked to As cycle, Fe and S bacteria play a significant role in As mobilization in subsurface environments (Héry et al., 2008, 2015). S-oxidizing bacteria represented by *Sulfuricurvum* genus were abundant in the analyzed aquifers, whereas *Thiobacillus* and *Thiothrix* were retrieved in biofilter water. Sediment-associated elemental S and sulfides in the local aquifer may represent a source of electrons for these bacteria and might be responsible of dissolution of metals and metalloids associated to sulfide minerals. Recently, *Sulfuricurvum* and *Thiobacillus* were found to be predominant in sediment-associated bacterial communities in high As aquifers (Li P. et al., 2015). The contribution of S-oxidizing bacteria in S cycling has been recognized in subsurface environments, due to their metabolic versatility (Anantharaman et al., 2016). With this respect, *S. kujiense* (Kodama and Watanabe, 2004) as well as *Sulfuricurvum* strain RIFRC-1 (Handley et al., 2014) perform anaerobic and microaerobic oxidation of solid S as sulfide, elemental S and thiosulfate, also contributing to pyritic-S oxidation (Campbell et al., 2006). Different sulfate-reducing bacteria (SRB) were detected in all the samples, but with relative abundance always below 1%, probably because sulfate reduction is energetically less favorable and needs a lower redox potential than those measured in the studied sites (∼ -100 mV) (Emmerich et al., 2012). Similar results were obtained in Asian aquifers (Lee et al., 2011; Li et al., 2014). A significant implication of these results from the studied aquifer is that S bacteria could be responsible for the oxidative dissolution of Fe-S-As bearing sediments. Moreover, this trend did not seem to be mitigated by the presence of SRB, that are recognized to limit natural As contamination in groundwater (Kirk et al., 2004; Omoregie et al., 2013).

Fe-oxidizing bacteria *Rhodobacter* and *Leptothrix* were retrieved at a lesser extent, whereas *Gallionella*, a typical neutrophilic freshwater Fe-oxidizing bacterium was not retrieved. Fe-reducing bacteria like *Geobacter, Geothrix, Ferribacterium*, and *Desulfuromonas* were detected in samples A, I, D, and L. Among these, only *Geobacter* was abundant in site L (2.9%), possibly related to the presence of high Fe concentration in water. The other Fe-reducing bacteria accounted for less than 1% of the total community, thus suggesting that the contribution of this population to As release from sediments was limited, in accordance with Anantharaman et al. (2016). Members of the genus *Thiobacillus* have been shown to reduce Fe (Lovley, 2006). These organisms accounted for 5.4% in B-WW; however, the highly positive redox potential measured makes this metabolism unreliable at the site.

## Conclusion

The microbial communities of As-rich groundwaters of Northern Italy were characterized, revealing a wide diversity of uncultured bacterial species, thus deserving these environments of further attention. A complete microbial redox As cycle was present in most aquifers, according to the different As metabolic pathways retrieved by both molecular and cultivation approaches. In Northern Italy Pleistocene aquifers, differently from South and South East Asia aquifers, chemolithotrophy, mainly based on S cycle, might be involved in As mobilization processes. The retrievement of As(III)-oxidizing capacity in the microbiota represents an important source for future studies on the development of decontamination actions.

## Data Availability

The datasets generated for this study can be found in GenBank, sequences produced by pyrosequencing were deposited in the Genbank database within the BioProject PRJNA507727. Sequences for *aioA* genes were deposited under accession number MK497003-30, *cbbL* genes under accession number MK496989-7002, *arrA* genes under accession number MK415637-55, DGGE bands under accession number MK312575-88.

## Author Contributions

LC and GM contributed to conception and design of the study, to molecular and statistical analyses, and manuscript preparation. SZ and BA contributed to molecular analysis. PZ performed the chemical characterization of groundwater samples. MR and TB contributed to the hydrogeological setting of samples. All authors approved the submitted version.

## Conflict of Interest Statement

The authors declare that the research was conducted in the absence of any commercial or financial relationships that could be construed as a potential conflict of interest.
