# Supplementary material for: Exploring Biodiversity and Arsenic Metabolism of Microbiota Inhabiting Arsenic-Rich Groundwaters in Northern Italy
Source: Front Microbiol. 2019 Jul 2;10:1480. doi: 10.3389/fmicb.2019.01480 (PMC6614289; doi:10.3389/fmicb.2019.01480)
Supplement: Supplementary file 1 [file Data_Sheet_1.PDF]

Supplementary Material

1 Geographic location of sampled arsenic-contaminated aquifers

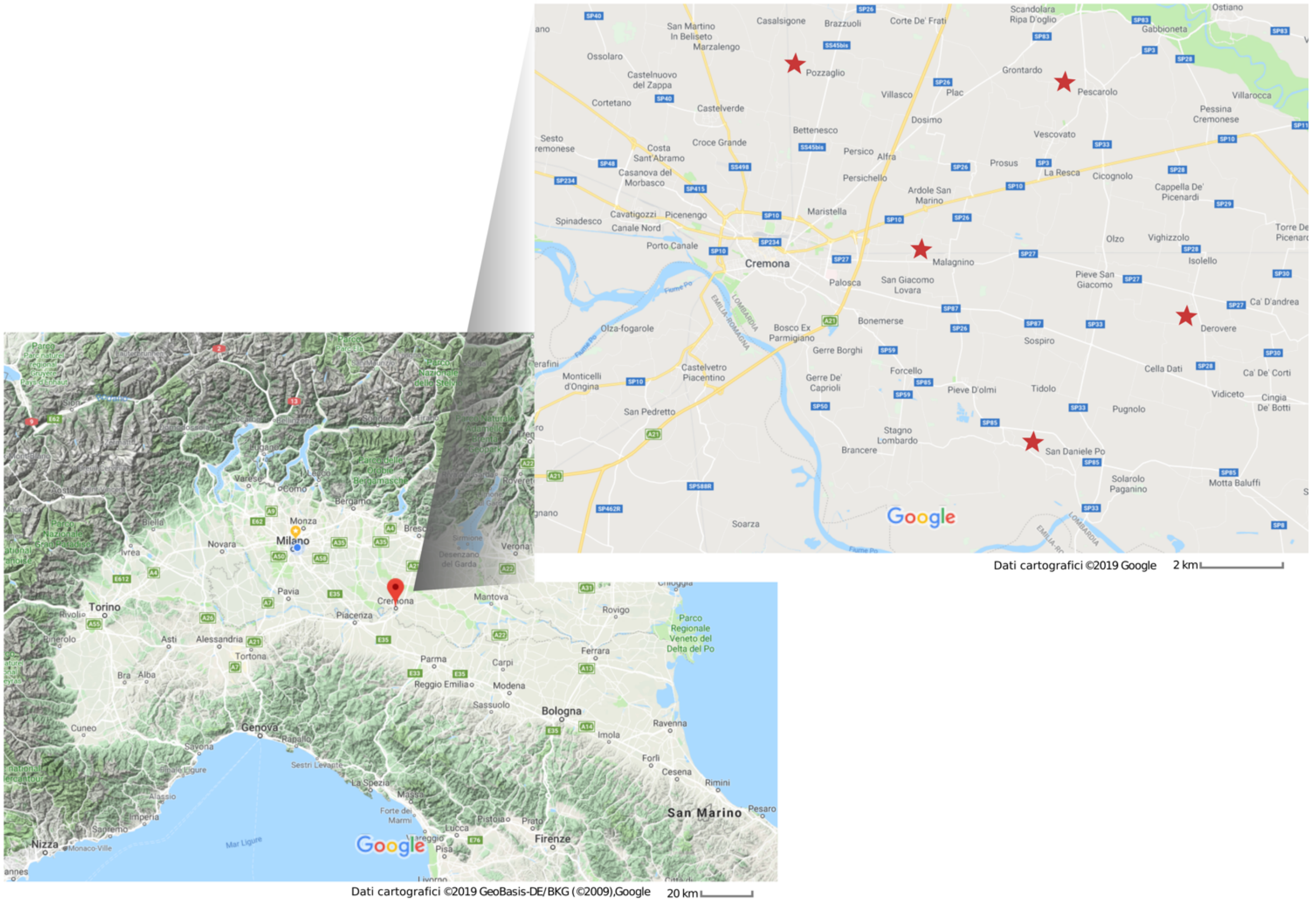

Supplementary Figure 1. Location of the sampling sites. Images adapted from Google Maps.

2 PCR-DGGE analysis of 16S rRNA genes

2.1 Experimental conditions

For denaturing gradient gel electrophoresis (DGGE) analysis, hyper variable portion of 16S rRNA was amplified from genomic DNA using primers Bac341fGC and Bac907rM which target the 16S rRNA gene of Bacteria (Schäfer and Muyzer, 2001). PCR reactions were performed in a final volume of 25 µL containing 1.25 µL of DNA template, 1x of Qiagen PCR Taq master mix (containing Taq-polymerase, dNTPs, buffer and stabilizing components) (Qiagen, Hilden, Germany), 0.5 µmol L^{-1} of

each primer. The amplification protocol used was the following: initial denaturation at 95°C for 5 minutes, 29 cycles at 95°C for 30 seconds, at 57°C for 40 seconds, at 72°C for 40 seconds, a final extension step at 72°C for 30 minutes and then kept at 4°C. Samples ready for the PCR analysis were put into the thermal cycler (T-Gradient, Biometra, Germany) when the first step of amplification protocol was completed. DGGE analysis of the PCR products was performed in a D-Code Universal Mutation Detection System (Bio-Rad, USA) apparatus and run in 20-70% denaturing gradient gel for 16 hours at 100 V with a constant temperature of 60°C. After completion of the electrophoresis the gels were incubated with SYBR Gold (Molecular Probes, USA) solution and documented with GelDoc System (Bio-Rad, USA). Bands were excised from the gels with a sterile scalpel, immersed in Milli-Q water for DNA elution and incubated overnight at 4°C. Re-amplification of the eluted DNA was performed with the same protocol using the same primers, but without the GC-clamp. DNA was sequenced using the Taq Dye-Deoxy Terminator Cycle Sequencing kit (Applied Biosystems, USA) with Bac907rM. Samples were run on a ABI 310 Genetic Analyser (Applied Biosystems). Analysis of DGGE banding patterns has been performed by Gelcompar II: Gel Electrophoresis Software (v5.0, Applied Maths). Similarity values were calculated using the Dice correlation coefficient and neighbor-joining method. The cluster cophenetic correlation was used to evaluate the branch quality of clusters. The number of visible bands in a DGGE profile was used as an indicator of alpha-diversity. Sequences obtained from DGGE bands were compared to sequences stored in the GenBank database by using BLASTN.

2.2 Results

DGGE of PCR-amplified bacterial 16S rRNA gene fragments revealed that groundwater community fingerprints were clustered into six groups at a 50% cut-off value (**Supplementary Figure 2**).

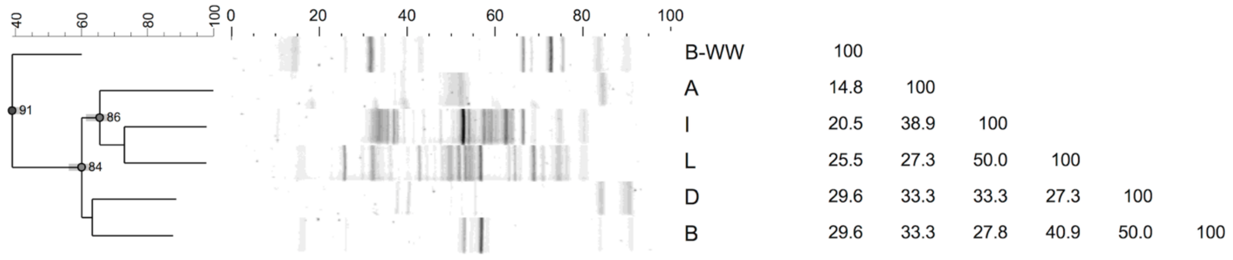

Supplementary Figure 2. Neighbor-joining cluster analysis of DGGE profiles of 16S rRNA gene fragments PCR-amplified from DNA extracted from the groundwater samples A, I, D, B, the monitoring well sample L, and the biofilter unit B-WW. Samples were assigned to clusters on basis of > 50% similarity (Dice coefficient). Nodes in the cluster indicate the cophenetic correlation.

According to neighbor-joining cluster analysis and the Dice coefficient, aerobic biofilter waters (B-WW) diverged from the other samples deriving from groundwaters characterized by negative redox potential (**Table 1**). Samples A, I and L on the one hand, and B and D on the other hand, formed two separate clusters. However, the chemical characteristics and the geographic collocation could not

explain this separation. The number of visible bands varied among the samples and was not correlated to As concentrations in groundwaters.

The identity of the predominant community members was inferred by comparative analysis of sequences from 35 excised DNA fragments and sequences stored in the GenBank database. Twenty-one sequences, aligned and imported in ARB to infer their phylogeny. Among the imported sequences, 15 OTUs were identified at the species level in the six samples, 11 of which had high percentage of similarities with uncultured bacteria (**Supplementary Tables 1 and 2**). To link these 16S sequences to functionally characterized species, sequences from uncultured microorganisms were excluded from the comparison to the GenBank database. With these settings, Betaproteobacteria was the dominant group (48%), followed by Nitrospirae (24%), Gammaproteobacteria (10%), Bacteroidetes (9%), Alphaproteobacteria and Spirochetes (5%) (**Supplementary Figure 3** and **Supplementary Table 1**).

Supplementary Table 1. Similarity percentages of nucleotide sequences retrieved from DGGE bands of groundwater DNA profiles including sequences from uncultured microorganisms.

Water Sample	DGGE band	accession	description	Max identity
A	2	FJ712609.1	Uncultured bacterium clone KZNMV-30-B39 16S ribosomal RNA gene, partial sequence	98
	3	FJ712609.1	Uncultured bacterium clone KZNMV-30-B39 16S ribosomal RNA gene, partial sequence	97
	4	EU746694.1	Uncultured bacterium clone TA3_10 16S ribosomal RNA gene, partial sequence	100
	28	CU926234.1	Uncultured Betaproteobacteria bacterium 16S rRNA gene from clone QEDN3BA01	98
	31	FJ712609.1	Uncultured bacterium clone KZNMV-30-B39 16S ribosomal RNA gene, partial sequence	98
B	35	DQ514574.1	Uncultured bacterium clone 16S10 16S ribosomal RNA gene, partial sequence	99
	5	FJ484986.1	Uncultured beta proteobacterium clone Z273MF91 16S ribosomal RNA gene, partial sequence	99
	6	HM584334.1	Uncultured bacterium clone BF2-47 16S ribosomal RNA gene, partial sequence	98
	32			
D	36	JN802219.1	Uncultured Nitrospira sp. clone 2A-24 16S ribosomal RNA gene, partial sequence	100
I	12	JN391811.1	Uncultured bacterium clone Q7462-HYBO 16S ribosomal RNA gene, partial sequence	99
	37	JN391811.1	Uncultured bacterium clone Q7462-HYBO 16S ribosomal RNA gene, partial sequence	99
L	17 w	DQ676353.1	Uncultured Bacteroidetes bacterium clone MVP-62 16S ribosomal RNA gene, partial sequence	99
	21	AB635912.1	Uncultured Spirochaetes bacterium gene for 16S ribosomal RNA, partial sequence, clone: DG-PN-E1	99
	16	JN183332.1	Uncultured Bacteroidetes bacterium clone W5-0h-30 16S ribosomal RNA gene, partial sequence	99
B-WW	26	JN802219.1	Uncultured Nitrospira sp. clone 2A-24 16S ribosomal RNA gene, partial sequence	100

Supplementary Table 2. Similarity percentages of nucleotide sequences retrieved from DGGE bands of groundwater DNA profiles excluding sequences from uncultured microorganisms.

Water Sample	DGGE band	accession	description	Max identity

**A**	2	CP000116.1	*Thiobacillus denitrificans* ATCC 25259, complete genome	93
	3	CP000116.1	*Thiobacillus denitrificans* ATCC 25259, complete genome	92
	4	HQ290507.1	Bacterium SCGC AAA018-N17 small subunit ribosomal RNA gene, partial sequence (*Leptothrix ochracea* Rhodocyclaceae)	97
	28	NR_041257.1	*Methylophilus methylotrophus* strain NCIMB 10515 16S ribosomal	97
	31	CP000116.1	*Thiobacillus denitrificans* ATCC 25259, complete genome	93
**B**	33	NR_043249.1	*Denitratisoma oestradiolicum* strain AcBE2-1 16S ribosomal RNA	95
	35	GU979422.1	*Candidatus* Magnetoovum mohavensis strain LO-1 16S ribosomal RNA gene, partial sequence	89
	5	AB552842.1	*Sulfuritalea hydrogenivorans* gene for 16S rRNA, partial sequence	97
	6	HQ290491.1	Bacterium SCGC AAA018-E6 small subunit ribosomal RNA gene, partial sequence (*Leptothrix ochracea* Rhodocyclaceae)	96
	32	NR_041681.1	*Sphingomonas japonica* strain KC7 16S ribosomal RNA	97
**D**	36	FP929003.1	*Candidatus* Nitrospira defluvii chromosome, complete genome	100
**I**	12	NR_044793.1	*Thiobacillus aquaesulis* 16S ribosomal RNA, complete sequence	94
	37	NR_044793.1	*Thiobacillus aquaesulis* 16S ribosomal RNA, complete sequence	93
	14	NR_044655.1	*Thiothrix unzii* strain A1; ATCC 49747 16S ribosomal RNA, complete	97
	15	NR_029287.1	*Nitrospira moscoviensis* strain NSP M-1 16S ribosomal RNA	98
**L**	17 w	AJ229217.1	Unidentified eubacterium from anoxic bulk soil 16S rRNA gene (clone BSV73)	93
	21	AY695841.1	Spirochaetes bacterium SA-10 16S ribosomal RNA gene, partial sequence	94
	16	AB623230.1	Bacteroidetes bacterium 4F6B gene for 16S ribosomal RNA, partial sequence	94
**B-WW**	25	NR_029287.1	*Nitrospira moscoviensis* strain NSP M-1 16S ribosomal RNA	97
	26	FP929003.1	*Candidatus* Nitrospira defluvii chromosome, complete genome	100
	27	NR_044655.1	*Thiothrix unzii* strain A1; ATCC 49747 16S ribosomal RNA, complete	98

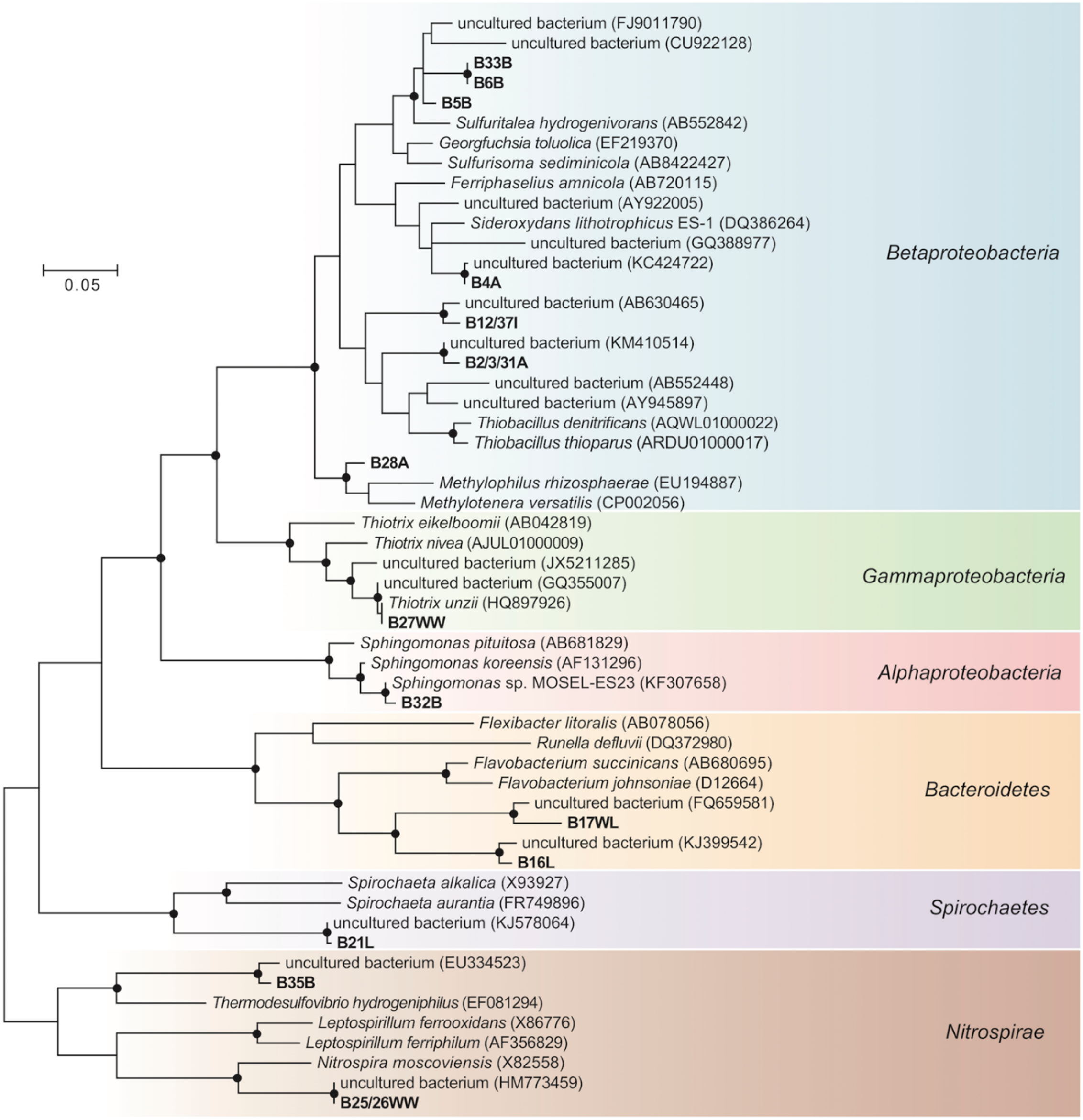

Supplementary Figure 3. Neighbor-joining phylogenetic tree of bacterial 16S rRNA gene sequences of bands retrieved from DGGE profiles of groundwater samples. The scale bar represents a difference of 0.05 nucleotides per position.

The majority of species revealed by DGGE analysis were likely resistant to As, having *arsC* gene in their genomes, while a minor fraction carried *aioA* and *arsM* genes. Notably, most of the sequences were related to sulfur-oxidizing bacteria like *Thiobacillus* spp., *Sulfuritalea hydrogenivirans*, *Thiothrix unzii* and *Candidatus* Magnetoovum mohavensis. The only Fe metabolism-related species was the Fe^{2+}-oxidizing bacterium *Leptothrix ochracea*. Nitrogen cycling was displayed by the presence of nitrite-oxidizing bacteria *Nitrospira moscoviensis* and *Candidatus* Nitrospira defluvii and the denitrifying bacteria *Denitratisoma oestradiolicum* and *Candidatus* Magnetoovum mohavensis. A sequence belonging to *Methylophilus methylophilus* was also detected, supporting cycling of one-carbon compounds in groundwaters.

3 16S rRNA gene pyrosequencing libraries

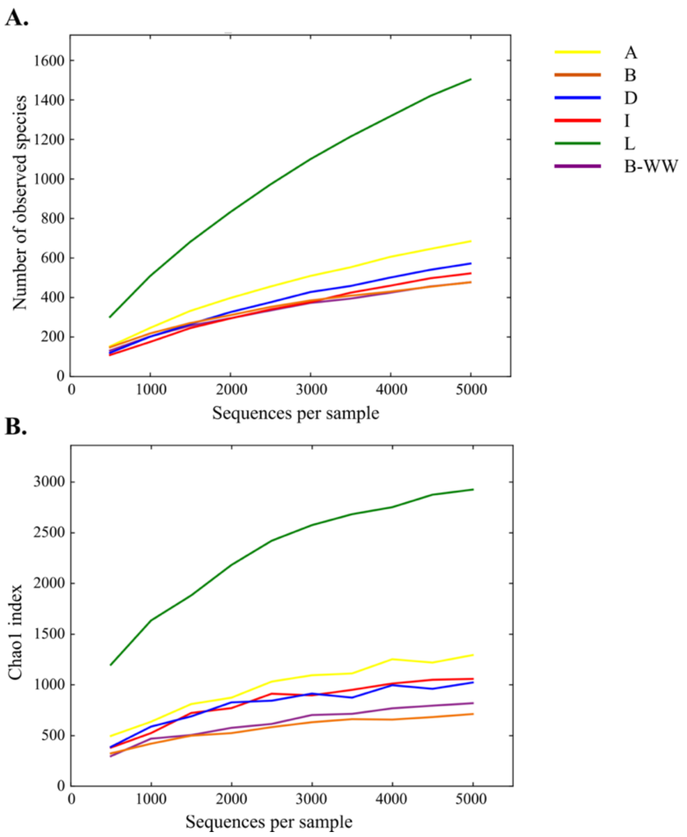

Supplementary Figure 4. Rarefaction analysis calculated for the number of species (A) and Chao1 index (B).

Supplementary Table 3. Average alpha diversity characteristics for drinking, monitoring and biofilter waters.

	OTUs (97%)[a]	Chao[b]	PD[c]
**A**	683.40	1291.612	39.243
**B**	475.90	816.713	27.056
**D**	520.90	1056.560	32.771
**I**	570.60	1020.996	36.763
**L**	1502.00	2924.816	80.178
**B-WW**	476.00	709.551	25.466

[a]Observed average OTUs after 5000 reads; [b]Chao1 average richness estimate (OUT 97%) after 5000 reads; [c]Pairwise distance average

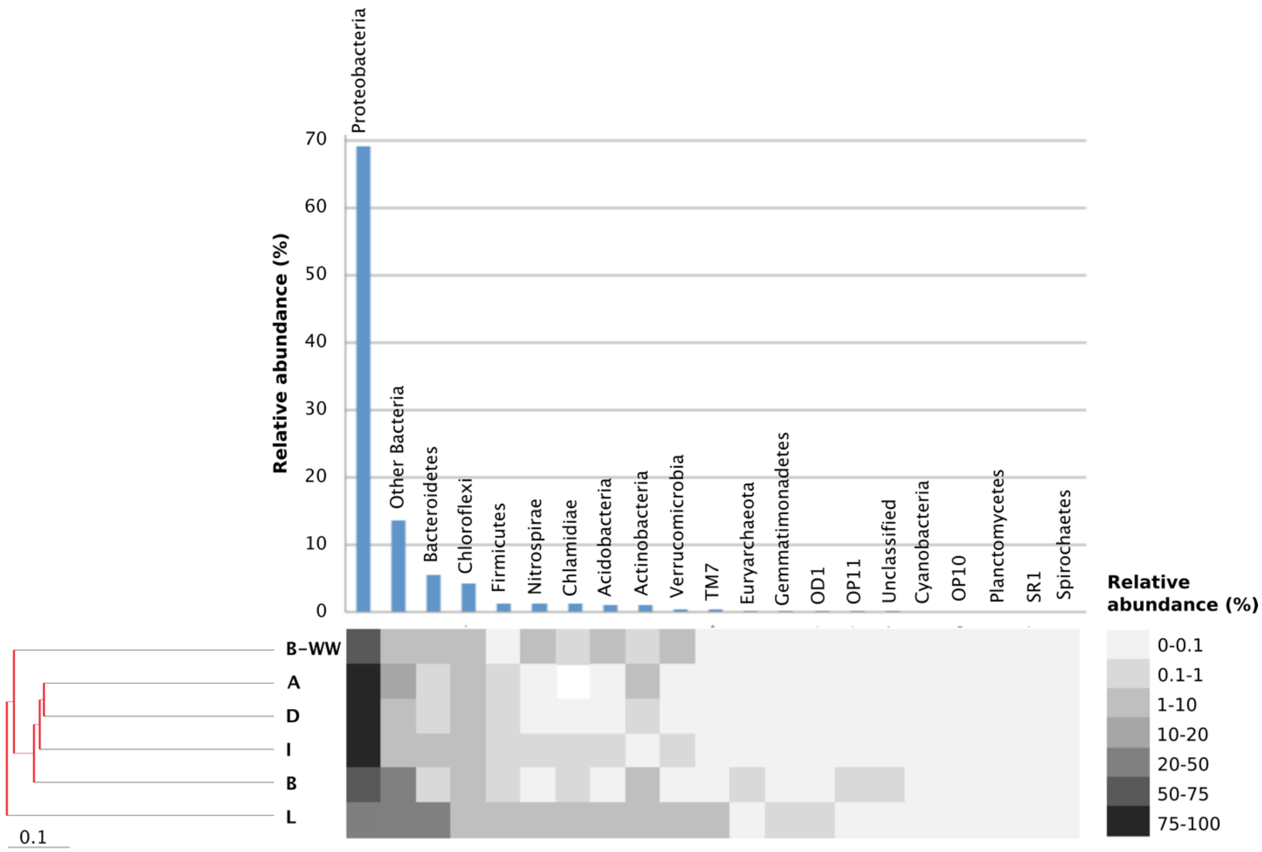

Supplementary Figure 5. UniFrac UPGMA cluster of 16S rRNA gene bar-coded sequences amplified in groundwater samples, bar graph showing the percentage abundance of 16S rRNA gene sequences of the core Phyla present in groundwater samples on the basis of 454 pyrosequencing data and heat map of different phyla from different sampling locations.

	DARB	ARB	AOB	FeRB	FeOB	SRB	SOB
A	0.17	6.98	6.67	0.40	0.93	0.21	0.16
B	0	16.96	1.15	0.18	0.86	0.43	15.11
D	0.19	75.70	25.35	0.28	16.32	0	50.39
I	0.13	67.86	1.55	0.98	0.96	0.02	66.91
L	3.39	9.11	3.31	3.67	3.51	0.18	2.62
B-WW	0	29.95	13.33	5.42	5.45	0	21.78

0 - 0.01
0.01 - 0.1
0.1 - 1
1 - 10
10 - 100

Supplementary Figure 6. Relative abundances of microorganisms involved in arsenic respiration (DARB), arsenic resistance (ARB), arsenite oxidation (AOB), iron reduction (FeRB), iron oxidation (FeOB), sulfur reduction (SRB) and sulfur oxidation (SOB). Values are reported as percentage (%).

Supplementary Table 4. Documented capacity of arsenic, iron and sulfur cycling in genera retrieved from the samples by 16S rRNA gene pyrosequencing.

Inferred function				Reference
Fe-red	Fe-ox	S-red	S-ox	
V	-	-	-	Achour et al 2007, Cavalca et al 2013, Heinrich-Salmeron et al 2011, Lovley
-	-	-	-	Kolek et al 2014, NCBI
-	-	-	-	NCBI
-	-	-	-	Lucker et al 2010
-	V	-	-	Byrne-Bailey et al 2010,, Cavalca et al
-	-	-	-	Giloteaux et al 2013, NCBI
-	-	-	-	NCBI, Cavalca et al 2013
-	-	-	-	NCBI
-	-	-	-	NCBI
-	-	V	-	Giloteaux et al 2013, Muyzer and
-	-	-	-	NCBI
V	V	-	V	NCBI, Lovley 2006, Hedrich et al
-	-	-	V	Meyer 2007, NCBI
-	-	-	V	Han et al 2012, Kodama 2004
V	-	V	-	Osborne et al 2015, Roden and Lovley
-	V	-	-	Achour et al 2007, Dubinina and
-	-	-	-	NCBI
V	-	-	-	Melton et al 2014, NCBI
V	V	-	-	Giloteaux et al 2013, Cavalca et al
V	-	-	-	Lovley 2006
-	-	-	-	Achour et al 2007, NCBI

Genus	As(V)-red	As(V)-res	As(III)-ox
Bacillus	V	V	V
Clostridium	-	V	V
Verrucomicrobium	-	V	-
Nitrospira	-	V	V
Acidovorax	-	V	V
Azotobacter	-	V	V
Hydrogenophaga	-	V	V
Nitrosospira	-	V	-
Nitrosomonadales	-	V	-
Desulfovibrio	-	V	-
Methanobacterium	-	V	-
Thiobacillus	-	V	V
Thiothrix	-	V	-
Sulfuricurvum	-	V	-
Desulfuromonas	V	V	-
Pseudomonas	-	V	V
Hyphomicrobium	-	V	V
Geothrix	-	V	-
Geobacter	V	V	-
Ferribacterium	-	-	-
Sphingopyxis	-	V	V

4 PCR-DGGE analysis on enrichment cultures

Supplementary Table 5. Affiliation of the DGGE bands separated from autotrophic As(III)-oxidizing enrichments cultures.

Sample	DGGE band	Acc. Number	Closest match in NCBI	Homology (%)
I	16	NR_024631	*Sphingopyxix chilensis* strain S37	97
	17	NR_026506	*Acidovorax defluvii* strain BSB411	99
	18	NR_026506	*Acidovorax defluvii* strain BSB411	91
	19	NR_024936	*Hydrogenophaga palleronii* strain CCUG 20334	98
L	20	NR_044250	*Lysobacter capsici* strain YC5194	99
	21	NR_043813	*Methyloversatilis universalis* strain FAM5	98
B-WW	26	NR_043478	*Exiguobacterium aurantiacum* strain DSM 6208	99

Supplementary Table 6. Affiliation of the DGGE bands separated from heterotrophic As(III)-oxidizing enrichments cultures.

Sample	DGGE band	Acc. Number	Closest match in NCBI	Homology (%)
I	38	NR_042851	*Rhizobium daejeonense* strain L61	98
	39	NR_042851	*Rhizobium daejeonense* strain L61	98
	40	NR_024786	*Delftia tsuruhatensis* strain T7	98
	41	NR_024786	*Delftia tsuruhatensis* strain T7	99
	42	NR_042851	*Rhizobium daejeonense* strain L61	98

L	43	NR_043254	*Chryseobacterium taeanense* strain PHA3-4	97
	44	NR_026209	*Acinetobacter lwoffii* DSM 2403	99
	45	NR_026209	*Acinetobacter lwoffii* DSM 2403	99
	46	NR_026506	*Acidovorax defluvii strain BSB411*	97
	47	NR_041588	*Variovorax boronicumulans* strain BAM-48	95
	48	NR_024709	*Pseudomonas hibiscicola* strain ATCC 19867	98
	49	NR_024936	*Hydrogenophaga palleronii* strain CCUG 20334	99
B-WW	51	NR_044343	*Luteimonas astuarii* strain B9	97
	52	NR_043007	*Hoeflea marina* strain LMG 128	97

Supplementary Table 7. Affiliation of the DGGE bands separated from As(V)-resistant enrichments cultures.

Sample	DGGE band	Acc. Number	Closest match in NCBI	Homology (%)
B-WW	55	NR_024631	*Sphingopyxix chilensis* strain S37	97
	56	NR_026506	*Acidovorax defluvii* strain BSB411	98
	57	NR_026506	*Acidovorax defluvii* strain BSB411	97
	58	NR_044343	*Luteimonas astuarii* strain B9	98
	59	NR_043813	*Methyloversatilis universalis* strain FAM5	97
	60	NR_026185	*Rhodococcus ruber* strain DSM43338	99
	62	NR_042263	*Microbacterium hydrocarbonoxydans* strain DSM 16089	99
	64	NR_026506	*Acidovorax defluvii* strain BSB411	99

5 References

Achour, A.R., Bauda, P., and Billard, P. (2007). Diversity of arsenite transporter genes from arsenic-resistant soil bacteria. *Res. Microbiol.* 158,128-137. doi:10.1016/j.resmic.2006.11.006

Byrne-Bailey, K. G., Wrighton, K. C., Melnyk, R. A., Agbo, P., Hazen, T. C., and Coates, J. D. (2010). Complete genome sequence of the electricity-producing "*Thermincola potens*" strain JR. *J. Bacteriol.* 192, 4078-4079. doi:10.1128/JB.00044-10

Cavalca, L., Corsini, A., Zaccheo, P., Andreoni, V., and Muyzer, G. (2013). Microbial transformations of arsenic: perspectives for biological removal of arsenic from water. *Future Microbiol.* 8, 753-768. doi:10.2217/fmb.13.38

Dubinina, G.A., and Sorokina, A.Y. (2014). Neutrophilic lithotrophic iron-oxidizing prokaryotes and their role in the biogeochemical processes of the iron cycle. *Microbiology* 83, 1-14. doi:10.1134/S0026261714020052

Giloteaux, L., Holmes, D.E., Williams, K.H., Wrighton, C.K., Wilkins, M.J., Montgomery, A.P., et al. (2013). Characterization and transcription of arsenic respiration and resistance genes during in situ uranium bioremediation. *ISME J.* 7, 370-383. doi:10.1038/ismej.2012.109

Han, C., Kotsyurbenko, O., Chertkov, O., Held, B., Lapidus, A., Nolan, M., et al. (2012). Complete genome sequence of the sulfur compounds oxidizing chemolithoautotroph *Sulfuricurvum kujiense* type strain (YK-1^T). *Stand. Genomic Sci.* 6, 94-103. doi:10.4056/sigs.2456004

Hedrich, S., Schlömann, M., and Johnson, D.B. (2011). The iron-oxidizing Proteobacteria. *Microbiology* 157, 1551-1564. doi:10.1099/MIC.0.045344-0

Heinrich-Salmeron, A., Cordi, A., Brochier-Armanet, C., Halter, D., Pagnout, C., Abbaszadeh-fard, E. et al. (2011). Unsuspected diversity of arsenite-oxidizing bacteria as revealed by widespread distribution of the *aoxB* gene in Prokaryotes. *Appl. Environ. Microbiol.* 77, 4685-4692. doi:10.1128/AEM.02884-10

Kodama, Y., and Watanabe, K. (2004). *Sulfuricurvum kujiense* gen. nov., sp. nov., a facultatively anaerobic, chemolithoautotrophic, sulfur-oxidizing bacterium isolated from an underground crude-oil storage cavity. *Int. J. Syst. Evol. Microbiol.* 54, 2297-2300. doi:10.1099/ijs.0.63243-0

Kolek, J., Sedlář, K., Provazník, I., and Patáková, P. (2014). Draft genome sequence of *Clostridium pasteurianum* NRRL B-598, a potential butanol or hydrogen producer. *Microbiol. Resour. Announc.* 2, e00192-14. doi:10.1128/genomeA.00192-14

Lovley, D. (2006). Dissimilatory Fe(II)- and Mn(IV)-reducing prokaryotes. *Prokaryotes* 2, 635-658. doi:10.1007/0-387-30742-7_21

Lücker, S., Wagner, M., Maixner, F., Pelletier, E., Koch, H., Vacherie, B., et al. (2010). A Nitrospira metagenome illuminates the physiology and evolution of globally important nitrite-oxidizing bacteria. *Proc. Nat. Acad. Sci.* 107, 13479-13484. doi:10.1073/pnas.1003860107

Malasarn, D., Saltikov, C.W., Campbell, K.M., Santini, J.M., Hering, J.G., and Newman, D.K. (2004). *arrA* is a reliable marker for As(V) respiration. *Science* 306, 455. doi:10.1126/science.1102374

Melton, E.D., Swanner, E.D., Behrens, S., Schmidt, C., and Kappler, A. (2014). The interplay of microbially mediated and abiotic reactions in the biogeochemical Fe cycle. *Nat. Rev. Microbiol.* 12, 797-808. doi:10.1038/nmrmicro3347

Meyer, B., Imhoff, J.F., and Kuever, J. (2007). Molecular analysis of the distribution and phylogeny of the soxB gene among sulfur-oxidizing bacteria – evolution of the Sox sulfur oxidation enzyme system. *Environ. Microbiol.* 9, 2957-2977. doi:10.1111/j.1462-2920.2007.01407.x

Muyzer, G., and Stams, A.J. (2008). The ecology and biotechnology of sulphate-reducing bacteria. *Nat. Rev. Microbiol.* 6, 441-454. doi:10.1038/nrmicro1892

Osborne, T.H., McArthur, J.M., Sikdar, P.K., and Santini, J.M. (2015). Isolation of an arsenate-respiring bacterium from a redox front in an arsenic-polluted aquifer in West Bengal, Bengal Basin. *Environ. Sci. Technol.* 49, 4193-4199. doi:10.1021/es504707x

Pfenning, N., and Biebl, H. (1976). *Desulfuromonas acetoxidans* gen. nov. and sp. nov., a new anaerobic, sulfur-reducing, acetate-oxidizing bacterium. *Arch. Microbiol.* 110, 3-12. doi:10.1007/BF00416962

Roden, E. E., and D. R. Lovley. 1993. Dissimilatory Fe(III) reduction by the marine microorganism, *Desulfuromonas acetoxidans*. *Appl. Environ. Microbiol.* 59, 734–742.

Schäfer, H., and Muyzer, G. (2001). Denaturing gradient gel electrophoresis in marine microbial ecology. *Methods Microbiol.* 30, 425-468. doi:10.1016/S0580-9517(01)30057-0
